# Solution Crystallization of Polycarbonate Surfaces for Hydrophobic State: Water Droplet Dynamics and Life Cycle Assessment towards Self-Cleaning Applications

**DOI:** 10.3390/polym13091449

**Published:** 2021-04-30

**Authors:** Bekir Sami Yilbas, Abba Abdulhamid Abubakar, Hussain Al-Qahtani, Shahzada Zaman Shuja, Mian Mobeen Shaukat, Ahmet Z. Sahin, Abdullah Al-Sharafi, Saeed Bahatab

**Affiliations:** 1 Mechanical Engineering Department, King Fahd University of Petroleum and Minerals (KFUPM), Dhahran 31261, Saudi Arabia; abba.abubakar@kfupm.edu.sa (A.A.A.); qahtanih@kfupm.edu.sa (H.A.-Q.); shuja@kfupm.edu.sa (S.Z.S.); mshaukat@kfupm.edu.sa (M.M.S.); azsahin@kfupm.edu.sa (A.Z.S.); alsharafi@kfupm.edu.sa (A.A.-S.); g201074560@kfupm.edu.sa (S.B.); 2 Center of Research Excellence in Renewable Energy (CoRE-RE), King Fahd University of Petroleum and Minerals (KFUPM), Dhahran 31261, Saudi Arabia; 3 K.A.CARE Energy Research & Innovation Center, Dhahran 31261, Saudi Arabia

**Keywords:** polycarbonate, solution treatment, hydrophobicity, silicon oil, dust removal

## Abstract

Polycarbonate sheets are optically transparent and have the potential to be used as one of the cover materials for PV applications. Solution treatment of polycarbonate surfaces enables to create surface texture topology giving rise to a hydrophobic state, which is favorable for self-cleaning applications. In the present study, hydrophobization of polycarbonate surface is investigated via crystallization of surface by a one-step process. The influence of texture topology, which is created via crystallization, on water droplet mobility and optical transmittance is examined. Findings revealed that solution treatment, using acetone, results in crystallized polycarbonate surfaces with a hydrophobic state. Depending on the treatment duration, the texture characteristics of crystallized surface change while influencing the water contact angle hysteresis. This in turn affects the droplet mobility over the inclined crystallized surface and alters the UV visible transmittance. Moreover, the droplet mobility improves and dust mitigation rates from the treated surface increase as the solution treatment duration are reduced to 2 min. Oil impregnated samples result in improved UV visible transmittance; however, droplet motion changes from rolling to sliding over the surface. A sliding water droplet enables the removal of the dust particles from the oil-impregnated sample surface.

## 1. Introduction

The use of renewable energy sources becomes critically vital in the overall energy production portfolio. Solar energy is the abandoned renewable energy resource, which can be utilized, mainly, in two forms heat and electricity. In this regard, solar thermal power and photovoltaic applications are widely considered harvesting solar energy. One of the challenges for photovoltaic applications is to produce electricity with high efficiency at minimum cost. Although significant research studies towards the improvement of photovoltaic efficiency have been reported, the effect of harsh environments such as dust and high temperature on the operational efficiency of such devices is still in progress. The vast changes in climate enhance the dust storm cycles, which contribute to the degradation of photovoltaic outputs [1]. In general, small size dust and airborne particles can settle over the photovoltaic panel surfaces while creating an intermediate layer between solar optical radiation and the panel surface. Accumulated particles can cause scattering and absorption of UV visible radiation reaching the panel surface while preventing efficient electricity production from the panel. However, highly transmitting hydrophobic panels can minimize the dust effects via lowering dust adhesion over the surfaces. On the other hand, polycarbonates (PC) panels have high optical transmittance and they are extensively used in industry for several purposes because of their low cost. Polycarbonate is derived from bisphenol A (BPA) during reaction with phosgene COCl_2_ and it consists of thermoplastic polymers with carbonate groups. Although PC panels have strong and flexible mechanical characteristics, high surface free energy causes the strong attachment of small dust particles over the surfaces, i.e., the efforts required to mitigate dust from PC panel surfaces remain considerably high. Hydrophobization of PC surfaces can lower dust adhesion over the surfaces despite the fact that optical transmittance can be lowered due to hydrophobic texture topology, which results in the specular and diffusive scattering of incident UV visible radiation. In addition, the one-step hydrophobicity of PC surfaces becomes demanding because of the low cost. Consequently, the development of one-step hydrophization of PC surfaces with high optical transmittance becomes fruitful for practical photovoltaic applications.

Surface hydrophobization gives rise to surface texture having low scale hierarchically dispersed pillars and cavities over the surface. The typical texture patterns possess micro and sub-micro pillars. Creating such patterns on PC surfaces requires physical/chemical or both physical and chemical modifications of the surfaces. The solution crystallization of PC surfaces is one of the possible candidates resulting in such texture patterns over the surface. Polycarbonate can be crystallized using the acetone immersion technique because of its molecular chain flexibility [2]. Several studies have reported the crystallization of polycarbonate surfaces [3,4,5]. Producing the crystallized polymeric materials via incorporating nanoparticles, such as carbon nanotubes, can generate a hydrophobic state on the surface [3]. In some applications of sensing technologies, bonding of polymeric hydrophobic surface with the sensing chip becomes critical for practical applications. One-step process of bonding such chips to a hydrophobic polymeric material becomes important for microfluidic applications [5]. Furthermore, in microfluidic applications, the method of hydrophobization of polycarbonate via solution treatment becomes important. This is because of the fact that surface roughness of the microchannels must be sufficiently low for efficient microfluidic applications. Hence, the proper settings of the processing parameters such as exposure duration and acetone concentration are vital towards achieving the desired surface roughness [6]. The surface roughness of micropatterned polymer, in terms of average pillar heights, becomes vital for creating a amphiphobicity surface for water droplet condensation and fluid motion in the microchannels [7]. In medical applications, the use of crystalline polycarbonate in self-assembly for drug release provides a controllable mechanism of delivery [8]. In addition, hydrophobic polycarbonate can be used towards producing stable and biocompatible surfaces. In this case, dodecylamine can be used to initiate the reaction with carbonate groups of polycarbonate producing a hydrophobic state on the surfaces [8]. Moreover, polycarbonate degradation and stability are affected by the crystallization process. Surface crystallization does not only create a hydrophobic state, but it also increases the rate of elongation at break as compared to amorphous polycarbonate during the hydrolysis. In addition, the impact strength and density reduce as polycarbonate is crystallized; however, the chemical stability of the crystallized surface remains higher than the amorphous structure when subjected to hydrolysis [9]. On the other hand, high power beams, such as lasers, can be used to texture polycarbonate surfaces towards achieving the hydrophobic states [10,11,12]. In general, laser texturing gives rise to hierarchical micro/nanopatterns over the surface, which form the bases for creating the hydrophobic states. However, laser textured surfaces have limitations in optical transmittance and uniformity of wetting states over the treated surface. Laser treatment, also, remains costly because the high energy beam ablation is involved despite its precision of operation. Solution treatment for texturing of polycarbonate surface is favorable over the other methods because of the low cost and short processing time (one-step process). Surface texture characteristics such as height of pillars and their distribution can be controlled through proper setting of the crystallizing parameters [4].

On the other hand, sustainable manufacturing processes related to the solution treatment are important to fight the rising threat of climate change. Usually, the environmental impacts of novel manufacturing processes are not studied until the processes are adopted at a large scale and become widely used. At this stage, it is beneficial to examine the environmental impacts of novel processes as it can help to identify the potential environmental burdens these processes are producing. Furthermore, life cycle analysis can also help to pinpoint those materials and resources, which are contributing to a significant percentage of the environmental impacts. Life cycle assessment is the most commonly and extensively used technique to quantify the environmental impacts of products and processes [13]. The life cycle assessment method identifies the potential impacts of a product throughout its entire life span. In addition, life cycle analysis provides very detailed information about various environmental impacts generated by each stage of the product life cycle and helps to avoid shifting of impacts from one life cycle stage to another [14].

Mechanical flexibility and high optical transmittance of polycarbonate sheets enable them to be used in various applications. Although hydrophobicity of polycarbonate surfaces via solution treatment is examined previously [2,15], the main focus is to evaluate the texture characteristics of the resulting surfaces. The adhesion of dust particles and the assessment of water droplet mobility on the crystallized surface are left for future studies. In addition, surface texturing through crystallization lowers the UV visible transmittance of the crystallized surface, which limits the applications in photovoltaic panels. Consequently, the present study investigates the solution crystallization of polycarbonate wafers towards evaluating water droplet mobility and UV visible transmittance of the crystallized surfaces. The texture characteristics, such as globules and fibrils structures, are analyzed via analytical tools. The mechanical properties of the crystallized surface, in terms of scratch resistance and hardness, are evaluated. Water droplet motion on the crystallized surface is determined using the high-speed recording facility and the self-cleaning capability of the crystallized surface is evaluated. The UV visible transmittance of oil impregnated crystallized samples is explored. In addition, the life cycle assessment of the process parameters related to the solution crystallization of polycarbonate wafer are presented.

## 2. Experimental and Life Cycle Model

### 2.1. Experimental

Polycarbonate, derived from bis(phenol-A), wafers were used preparing samples in 1.5 × 30 × 80 mm^3^ (thickness, width, and length). Acetone solutions at different concentrations ranging between 30% and 60% (volume fraction) were used to crystallize the sample surfaces. The acetone solution temperature was kept at 298 K during the crystallization of the samples. The temperature setting was critical to achieve almost identical and repeatable texture characteristics of the crystallized surfaces. A fixture was designed and realized immersing the sample into an acetone bath while ensuring crystallization of one surface of the sample. The bathing duration was limited within 2–6 min range in accordance with the early study [15]. The initial findings revealed that acetone concentration of 30% resulted in the crystalized surface having hierarchically structured pillars with micro and nano topologies. Once the samples were taken out from the acetone bath, surfaces were cleaned with desalinated water jet at low velocity (0.5 m/s jet velocity). Later, crystallized sample surfaces were left dry in air ambient. The resulting texture topology of the sample surfaces was analyzed using electron scanning (Jeol 6460) and atomic force (Flex-Axiom, Nanosurf) microscopes. The infrared spectrum of absorption/emission of the crystallized surface was evaluated via Fourier transform infrared spectroscopy (Nicolet Nexus 670 FTIR). The crystal structures were analyzed using an X-ray diffractometer (XRD, Bruker D8 advance). The wetting states on the textured surfaces were assessed by a drop contact angle measurement method utilizing a goniometer (Kyowa, model-DM 501). In contact angle measurements, a high-precision shape analysis was accommodated [16]. In this case, the contact angle measurements were carried out using the optical refection of droplet shape appearing on the Goniometer screen. Since the actual droplet shape was spherical and optical image appeared to be in two-dimensional circular shape, some small errors could occur on the two-dimensional optical image of the droplet (because of optical distortion). Nevertheless, this error could be minimized via proper adjustment of the light illumination source and the Goniometer camera. Once the image was captured, a function for the semicircle was developed using the Gaussian curve fitting algorithm. This arrangement enabled to obtain droplet geometric feature of the droplet in the functional form with best fitting accuracy. Since the contact angle measurement was carried out along the three-phase contact line (triple points), the horizontal positioning of the reference-line coinciding with the contact line remained critical in high precision droplet shape analysis (HPDSA). In some cases, a small shift of reference line (*α_BL_*) from the horizontal line could occur. This needs to be incorporated during the contact angle assessments from the best curve fitting function. Hence, the contact angle of the hydrophobic droplet (>90°) obtained from the best fitted function took the form [16]:

(1) $$\theta =90^{o}+\arcsin\left(\frac{\Delta y}{R}\right)\mp \alpha_{BL}$$

where, *α_BL_* is the inclination angle of the baseline (for correcting the non-horizontal baseline) and ∆*y* is the difference in height coordinates between the triple points and the center of the fitting circle. *R* is the of the curvature of the image when best fitted in to a function.

The average values of contact angle and hysteresis are provided in the table (Table 1) after 12 repeats of contact angle measurements at different locations on the surface. In addition, contact angle measurements are repeated for different samples prepared under the same crystallization conditions. The standard deviation of the measurements based on the different samples is about 3°. The UV visible transmittance of the treated (crystallized) samples was assessed through UV visible spectrometer (67 Series spectrophotometer, Jenway). UV visible transmittance of 6 min treated dry and oil impregnated samples were measured. Since the crystallized surface scatters and partially absorbs the incoming optical spectrum, it demonstrates the partial opaque feature. In order to improve UV visible transmittance, the crystallized surfaces were impregnated by the film of silicon oil. The selection of silicon oil was due to its refractive index (1.405), which was close to polycarbonate (1.59). Silicon oil had a viscosity of 10 cst, density of 935 kg/m^3^, and surface tension of 35 mN/m. Silicon oil film on impregnated sample surfaces was measured by an ellipsometer (M-2000 J.A. Woolam Co., Lincoln, NE, USA) and the oil film thickness of 700 µm was achieved over the crystallized surface. A high-speed camera (Dantec, SpeedSense 9040, Skovlunde, Denmark) and a tracker program were utilized evaluating the water droplet behavior on crystallized as well crystallized-oil-impregnated surfaces. The high-speed camera was operated for 5000 fps with a resolution of 1280 × 800 pixels having size of 14 × 14 µm^2^. Based on the multiple records of droplet motion, the experimental error was evaluated as 3%. A new fixture was also designed and realized for dust removal experiments from the treated surfaces. In this case, environmental dust was gathered from PV panel surfaces and uniformly deposited over the sample surfaces. The optical camera system was used to evaluate dust residues along the rolling and sliding water droplet paths over the dusty sample surfaces. The pinning force acting on the droplet over the hydrophobic surface is related to the receding and advancing angles of the droplet, which takes the form [17]:

(2) $$F_{ad}=\frac{24}{\pi^{3}}\gamma fD_{H}\left(cos\theta_{R}-cos\theta_{A}\right)$$

here, γ is the surface tension, *f* is the solid fraction, *D_H_* being the wetting diameter. It is worth mentioning that the solid fraction is a similar order of roughness parameter. Hence, introducing advancing and receding angle of the droplet given in Table 1, using solid fraction for 2 and 6 min of treatment (f = 0.58 and f = 0.52 for 6 and 2 min treatments), and the wetting diameters are about 2.1 and 2.2 mm for 6 and 2 min treatment, respectively. This leads the adhesion forces about 3.83 × 10^−7^ N and 2.6 × 10^−7^ N for 6 and 2 min treatments, respectively. The scratch resistance of the treated surfaces is evaluated according to the ASTM D7027-05 standard [18]. During the scratch tests, the contact load and end load of the indenter are set as 0.03 and 5 N, respectively. The scratch hardness is evaluated from: $H_{s}=\frac{4qP}{\pi w^{2}}$, here *P* is the applied load (in N), *w* being the width of scratch path (in mm), and *q* is the constant depending on the elastic recovery.

### 2.2. Life Cycle Model

The impact assessment methods involved four steps, which included goal and scope definition, life cycle inventory compilation, life cycle assessment, and interpretation of results (ISO, 2006). The life cycle analysis started with a clear and defined scope of the tasks, functional unit, and system boundaries. The life cycle inventory comprised material and energy used in each step of the processes resulting in the final product, which was the crystallized polycarbonate surface. The inventory was, then, used to conduct the impact assessment and calculation of various environmental impact categories, such as global warming potential, ozone depletion potential, and similar [19]. The current goal of the life cycle assessment was to determine the environmental impacts of the solution crystallization process. The functional unit considered relied on the production of 1 m^2^ of crystalized polycarbonate surface. The analysis incorporated the life cycle stages of production of raw materials and the solution treatment process. The prorated data pertinent to the experiments for production 1 m^2^ of the crystallized surface was 2.25 L of acetone and 5.25 L of industrial grade water. Hence, these values were incorporated in the life cycle analysis. Since the main interest of the process was to produce crystalized hydrophobic polycarbonate surfaces, the cradle-to-gate life cycle analysis was incorporated. In this case, the analysis considered the life cycle of a product and process from resource extraction (cradle) to the condition that the product was available for practical usage. Hence, life cycle analysis was carried out using SimaPro software [20], which one of them is the leading software to conduct such studies. Moreover, ReCiPe method was selected as it an internationally accepted method and several life cycle impact studies have used this method [21].

## 3. Results and Discussion

### 3.1. Surface Crystallization and Texture Topology

**Figure 1:**
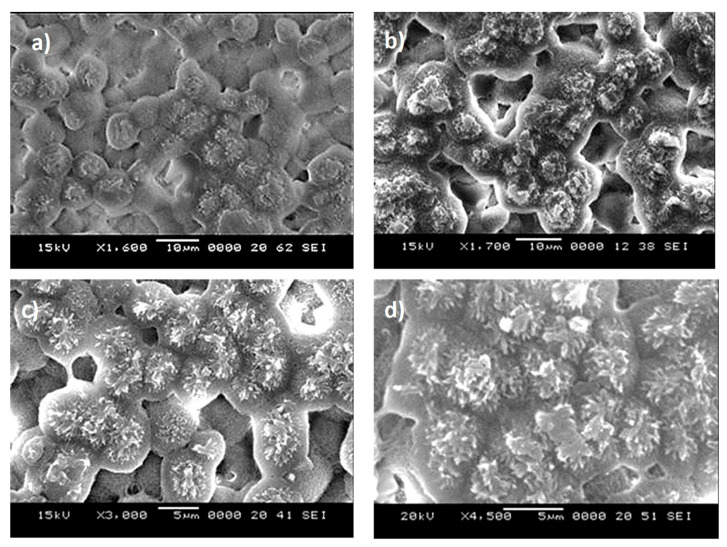
Scanning electron microscope (SEM) micro-images of solution treated polycarbonate: (**a**) spherules formed on sample surface after 2 min of treatment, (**b**) spherules formed on sample surface after 6 min of treatment, (**c**) fibrils emanating from spherules surface after 2 min of treatment, (**d**) fibrils emanating from spherules surface after 6 min of treatment.

**Figure 2:**
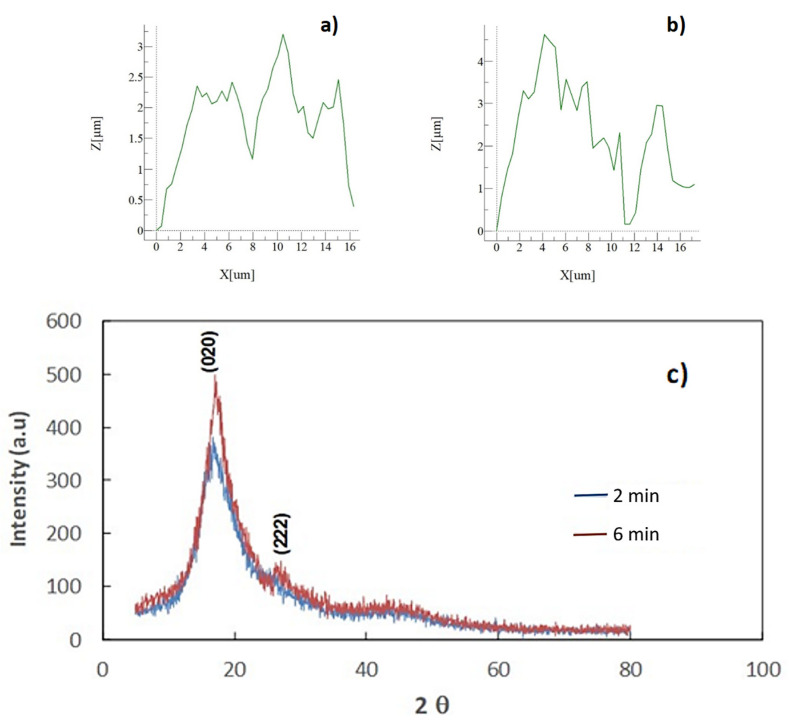
Atomic force microscopy line scan over treated surfaces: (**a**) line scan for 2 min treated surface, (**b**) line scan for 6 min treated surface, and (**c**) X-ray diffraction (XRD) data for crystallized treated surface for 2 and 6 min.

Figure 1a–d depicts SEM micrographs of solution crystallized surface for 6 and 2 min, respectively. The solution treatment of sample surfaces gives rise to surface topology having microspherules (Figure 1a,b) and fibrils (Figure 1c,d) of various sizes. The number of spherules on the projected area of the surface becomes less for 2 min of treatment duration than that corresponding to 6 min treatment. The variation of size and distribution of spherules on the surface is related to the crystallization period. The growth rate of crystals over the immersed surface depends on the number of potential sites for nucleation. As crystallization initiates, some crystal outlets extend from the nucleate site giving rise to connections of crystal sites over the surface, i.e., the intermittent branching is responsible for the connection of nearby spherules. However, some spherules demonstrate a premature formation on the surface and these spherules have a smaller size than that of the extended spherules. The solubility parameters of acetone (Hilbrand-solubility) are within the range of 20.1–20.3 J^1/2^cm^−3/2^ [22]. In the early period of crystallization of the polycarbonate surface, acetone solution penetrates into the substrate surface via diffusion while forming a swollen layer (gels-like layer). In addition, the diffusion causes the reduction of the glass-transition-temperature in the substrate behind the swollen layer. This in turn results in plasticizing of the gels-like layer [22]. The non-Fickian diffusion, which takes place across the swollen layer, can cause almost constant penetration velocity into the polycarbonate substrate [23]. As the crystallized sample is removed from the acetone bath, acetone evaporates from the sample surface causing temperature reduction in the surface vicinity. Hence, evaporation alters the glass-transition-temperature and causes supercooling of the swollen layer. Since the cooling rate over the surface is non-uniform, different sizes of spherules have resulted. It should be noted that acetone evaporation from the surface occurs transiently and results in locally scattered (non-uniform) nucleation sites. Moreover, the crystallization process can take place in three steps, which include initiation, primary, and secondary crystallization steps [24]. In the first step, the crystallization nucleus is initiated, and polymer chains aligned in parallel join up to the nucleation center. The crystal grows spontaneously towards reaching a critical size in the second phase [24]. In the third phase, bundles and lamellar structures are formed during the crystallization, providing that the size and type of crystals highly depend on the initial nucleus size and free energy normal to the direction of chains [25]. In addition, fibrils are formed over the spherules surface, which appear as whiskers emanating from spherules tops (Figure 1c,d). The formation of whiskers is more apparent for 6 min treated surfaces. This demonstrates that by keeping the treatment time longer, the size of fibrils increases. Moreover, the surface texture profiles of crystallized samples, which are obtained from an atomic force microscope, are shown in Figure 2a,b for 6 and 2 min solution treatment cycles. The spherules′ height and spacing differ for two crystallization cycles (2 and 6 min treatments). Hence, 6 min treatment results in almost 15% higher spherules height and 20% closer spherules spacing as compared to those resulted from 2 min treatment. The average surface roughness and roughness parameter changes for two treatment durations. The average surface roughness is about 2.2 μm for 2 min treatment while it is about 3.1 μm for 6 min

**Figure 3:**
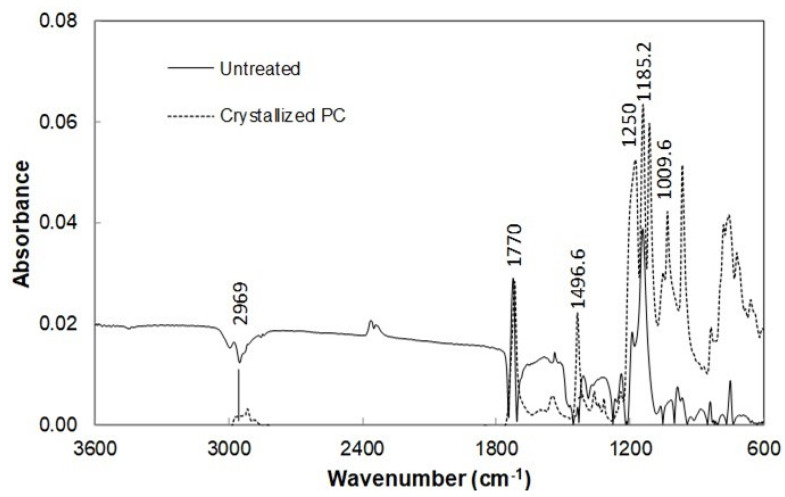
Fourier-transform infrared (FTIR) absorption data for crystallized and as received surfaces.

The roughness parameter represents the ratio of spherules area over the projected area of the surface. The roughness parameter for 6 min treatment is about 0.58 while it is about 0.52 for 2 min treatment. Figure 2c shows X-ray data for crystallized and received surfaces. The crystallinity of the treated layer is assessed through the X-ray diffraction (XRD) pattern for both treatment durations. XRD pattern for the solution treated surfaces demonstrates two diffraction peaks, which correspond to 020 phase and 222 phase. These peaks appear for both samples, which are treated for 2 and 6 min duration. The crystallinity is assessed via the ratio of total integrated intensities of XRD reflections due to crystalline peaks over the sum of the intensity of the scattered XRD reflections after subtraction of the background [26]. The crystallinity for 6 min treated sample is found to be about 31% while it is about 27% for 2 min treated sample, which is within the range of the early results [23]. FTIR spectrum obtained for the treated and as received polycarbonate surfaces are shown in Figure 3. In addition, the FTIR spectrum for the untreated surface is included in the figure for comparison. The treated surfaces for both treatment durations show peaks at 2874–2969 cm^−1^ absorption band, which are related to C-H bond-stretching-vibration [27]. The treated surfaces also have peaks at 860–680 and 1496 cm^−1^, which is attributed to C-H bending-vibration. It is worth mentioning that the bending vibration takes place at 860–680 cm^−1^ because of the C-H bond while the peak of 1496 cm^−1^ is due to the bending-vibration of the methylene group. The absorption peaks at 1700–1500 cm^−1^ are related to the bending vibration of the aromatic C=C bond. The absorption peak for stretching-vibration of ethers is observed at 1770 cm^−1^. Moreover, the mechanical properties including microhardness (Vickers hardness) and scratch resistance of the treated sample surfaces are evaluated. The surface microhardness is measured about 23.2 ± 0.8 HV for 6 min treated and 22.9 ± 0.8 HV for 2 min treated surfaces. The microhardness of the untreated sample surface is also measured for comparison, which is about 11.5 ± 0.4 HV.

Hence, crystallizing of samples enhances the hardness of the surface, which could demonstrate improved resistance to scratching as compared to untreated surface. The scratch resistance of the treated surfaces is evaluated incorporating the standard procedure (ASTM D7027-05) adopting the formulation: $H_{s}=\frac{4qP}{\pi w^{2}}$ and using *q* = 1 for elastic recovery and *q* = 2 is the case of surface deformation occurring during scratching [18]. The scratch hardness of as received sample is about 81 ± 4 MPa while it is 105 ± 4 and 115 ± 4 MPa for 2 and 6 min treated sample surfaces, respectively. Hence, the treated surfaces have higher scratch resistance than as received sample surface, which is more apparent for 6 min of treatment, i.e., the scratch resistance of the treated surfaces improves notably.

### 3.2. Hydrophobic Characteristics of Crystallized Surface and Water Droplet Mobility

The surface wetting states of the treated and as received surfaces are evaluated using the Goniometer via measuring the droplet dynamic contact angle. During the contact angle measurements, the method of high-precision drop shape analysis (HPDSA)) is accommodated [16]. It is worth mentioning that the contact angle of the surface depends on the texture topology and free energy of the textured surface. The Young’s equation provides the relation among the vapor, solid, and liquid interfacial surface tensions for the droplet [28]. However, the apparent contact angle provides a relatively realistic assessment of the wetting state for non-homogeneous surfaces [29]. The apparent contact angle on the surface ($\theta_{c}$) can be formulated via:

(3) $$cos\theta_{c}=f_{1}cos\theta_{1}+f_{2}cos\theta_{2}$$

where *f*_1_ represents the fraction of liquid–solid interface, *f*_2_ being the fraction of liquid–vapor interface on the surface, *θ*_1_ corresponds to the liquid–solid interfacial contact angle while *θ*_2_ is the liquid–vapor interfacial contact angle. Moreover, *f*_1_ can be represented as *f*, in terms of the solid fraction, which yields (1 − *f*). The value of *f* is within 0–1, i.e., *f* = 0 represents droplet is not contacting at the solid surface. Alternatively, *f* = 1 represents that the droplet fluid wets the surface completely.

**Figure 4:**
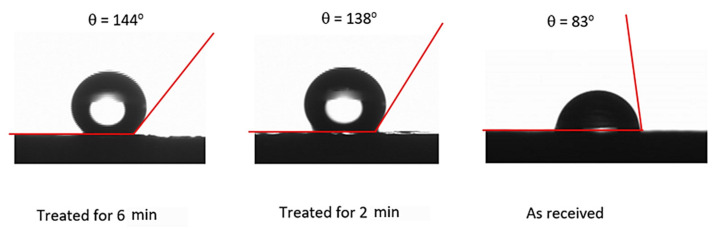
Optical image of droplet on 6 and 2 min treated surfaces, and as received surface. Images are obtained from Goniometer.

**Figure 5:**
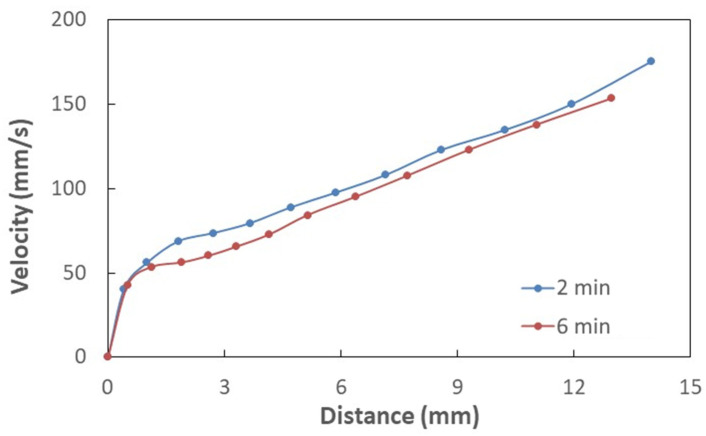
Water droplet translational velocity along 2 and 6 min treated surfaces. Droplet volume is 30 μL and inclination angle (δ) of treated surface is 10°.

**Figure 6:**
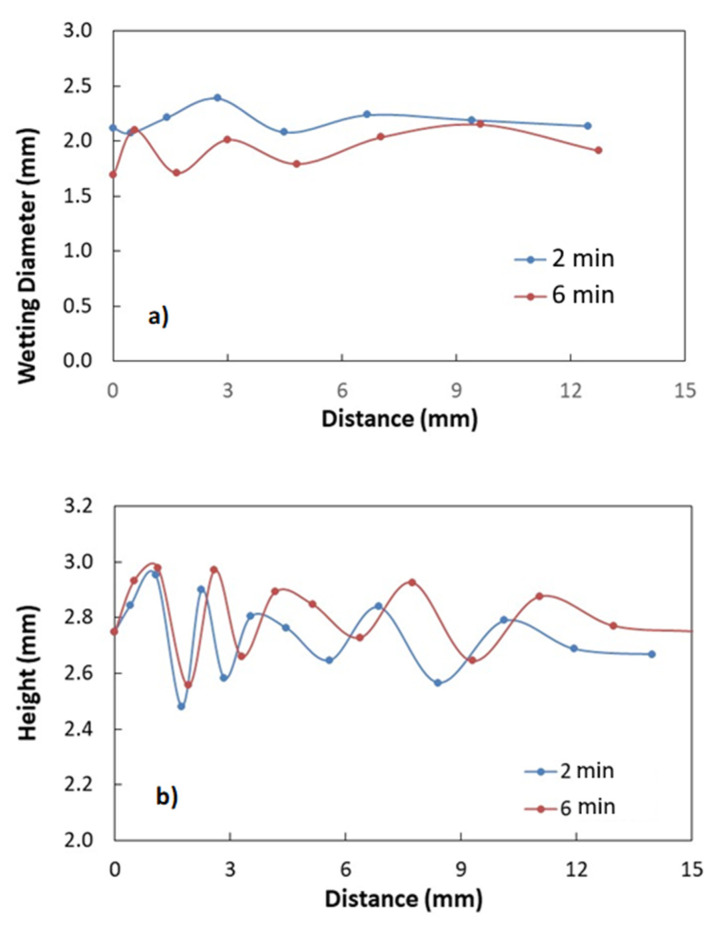
(**a**) Wetting diameter and (**b**) maximum height of the rolling droplet with distance over 2 and 6 min treated surfaces. Droplet volume is 30 μL and inclination angle (δ) of treated surface is 10°.

Table 1 gives the droplet contact angles together with hysteresis while Figure 4 shows the images of droplet on the treated and untreated surfaces. It is worth mentioning that the contact angle hysteresis is determined through the differences between advancing and receding angles of the droplet, i.e., *θ*_hysteresis_ = *θ*_Ad_ − *θ*_Rec_, here *θ*_Ad_ being advancing while *θ*_Rec_ is receding angles. Sample surfaces treated for 2 and 6 min result in hydrophobic state on the surface; however, as treated surface demonstrates hydrophilic state. The advancing and receding contact angles attain larger values for 6 min treatment than that corresponding to 2 min treatment despite the fact that the difference in contact angles is small for both surfaces. The solution treatment of the surface over longer period results in high pillar heights and closely spaced pillars over the sample surfaces. This enables to the improvement of the wetting state of the surface via high contact angle. However, contact angle hysteresis remains slightly larger for 6 min treated surface. Hence, increasing the solution treatment duration influences the contact angle hysteresis. It is worth mentioning that high contact angle hysteresis causes the liquid droplet pinning on the surface despite the fact that the surface has a high droplet contact angle. In order to evaluate the droplet pinning, further experiments were carried out. In this case, high-speed camera images of the droplet on the solution-treated sample surfaces were recorded. The water droplet pinning on the treated surface occurs because of the lateral (along the surface) component of the surface tension force opposing to the direction of droplet rolling under the gravitational influence [17,30]. The droplet pins the horizontal treated surface because of the adhesion force. As the treated surface is tilted about the horizontal plane, the droplet can roll and slip on the hydrophobic surfaces under the gravity. Hence, further experiments were realized to determine the droplet rolling speed along the inclined hydrophobic surface resulting from 6 and 2 min of treatments. In this case, high speed recorded data and tracker program were used to analyze the droplet translational velocity over the inclined hydrophobic surface. Figure 5 depicts the translational velocity of the droplet over the inclined hydrophobic surfaces that resulted from two different treatment periods. It is worth mentioning that the droplet volume and inclination angle of the surface are 30 and 10°. The experiments were repeated 12 times to ensure the droplet velocities as depicted in Figure 5. In this case, the experimental error, based on the high-speed data records, is in the order of 3%. The droplet volume and inclination angle were set same for both treated surfaces (6 and 2 min treatment duration). The droplet velocities over the hydrophobic surface change with time and the droplet velocity corresponding to the hydrophobic surface resulting from 2 min treatment becomes larger than that corresponding to 6 min treatment. This is because of the resisting (retarding) forces against the droplet rolling. The pinning, air drag, and shear forces oppose the droplet rolling under gravity. The shear force is created because of the interfacial shear between the droplet fluid and wetted surface across the droplet wetted area. Moreover, the droplet wobbles (puddles) under gravity [31]. The droplet puddle becomes less effective as the size of the droplet reduces to the capillary length ($\kappa^{-1}$), which takes the form: $\kappa^{-1}=\sqrt{\frac{\gamma_{LV}}{\rho g}}$, here *ρ* is droplet fluid density, and g is the gravitational acceleration [31]. The wobbling action causes alteration of the wetted area and the droplet′s maximum height. Figure 6a,b shows the droplet wetted diameter and the droplet′s maximum height along the inclined hydrophobic surface. The droplet wetted length slightly increases for 2 min treated surface compared to that of 6 min treated surface. The droplet maximum height remains almost the same for both treated surfaces, which is particularly true within the distance close to the droplet rolling initiation on the surface. This is because of the fact that same droplet volume is used for 2 and 6 min treated surfaces, In the early motion of the droplet, the influence of contact angle hysteresis (advancing and receding angle) becomes almost similar on the droplet motion. As the droplet motion continues, the difference in the maximum droplet height in between 2 and 6 min treated surfaces becomes large due to increasing contact angle hysteresis (Table 1). Hence, due to the droplet contact angle hysteresis (Table 1), the droplet translational velocity remains higher for 2 min treated surface than that of the 6 min treated surface (Figure 5) despite the fact that wetting diameter increases slightly for 2 min treated surface, i.e., low contact angle hysteresis ($\theta_{R}-\theta_{A}$) gives rise to smaller adhesion force despite *D_H_* increases slightly for 2 mm treatment duration in accordance to Equation (2). The total energy losses associated with the retarding forces for rolling droplet can be evaluated through the ratio of actual kinetic energy change of the droplet to the potential energy change over the hydrophobic surface. This ratio yields:

(4) $$\zeta =\frac{\frac{1}{2}\Delta V_{d}^{2}}{m_{d}g\Delta h}$$

here *V_d_* is the droplet translational velocity, m_d_ is the droplet mass and Δ*h* is the vertical height of the inclined hydrophobic surface (Δh=ΔLsinδ, here ΔL is the droplet pathlength on the hydrophobic surface and δ is the inclination angle of the hydrophobic surface from the plain surface). The ratio becomes about 0.25 and 0.20 for 2 and 6 min treated surfaces, respectively. Hence, the retarding forces for droplet rolling remains larger for 6 min treated hydrophobic surface. Although the droplet contact angle remains high for treated hydrophobic surface, high contact angle hysteresis gives rise to high loss of rolling droplet kinetic energy over the surface, particularly for 6 min treatment.

**Figure 7:**
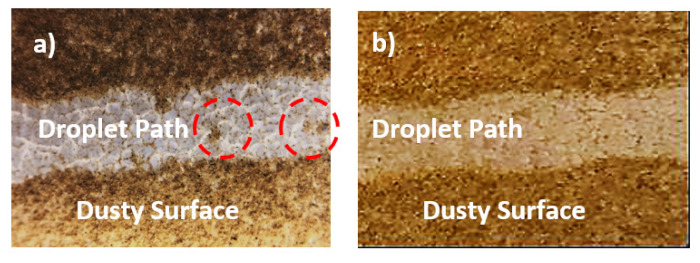
Optical view of droplet path over the dusty sample: (**a**) 2 min treated surface and red dotted regions shows the dust residues in the droplet path; (**b**) 6 min treated surface. Droplet volume is 30 μL and inclination angle (δ) of treated surface is 10°.

The dust particles mitigation from treated surfaces, by a rolling droplet, were also evaluated. In the dust mitigation experiments, 30 μL droplet and treated samples were inclined at 10° from the horizontal plane. It should be noted that the dust layer thickness was kept at about 300 μm and the dust layer was uniformly distributed over both treated surfaces. Figure 7 shows the droplet path from the dusty treated surfaces. The rolling droplet removes a slightly large amount of dust particles from both surfaces, which is more pronounced for 2 min treated surface. For 6 min treated surface, relatively large height of spherules may prevent droplet fluid from reaching the particles situated in between the spherules. This results in some dust residues remaining over the surface (Figure 7) for 6 min treated surface. Therefore, hydrophobic surface with low spherules heights has the advantage over the large spherule heights in terms of dust mitigation from the surface.

### 3.3. Improvement of Optical Transmittance of Crystallized Surfaces

**Figure 8:**
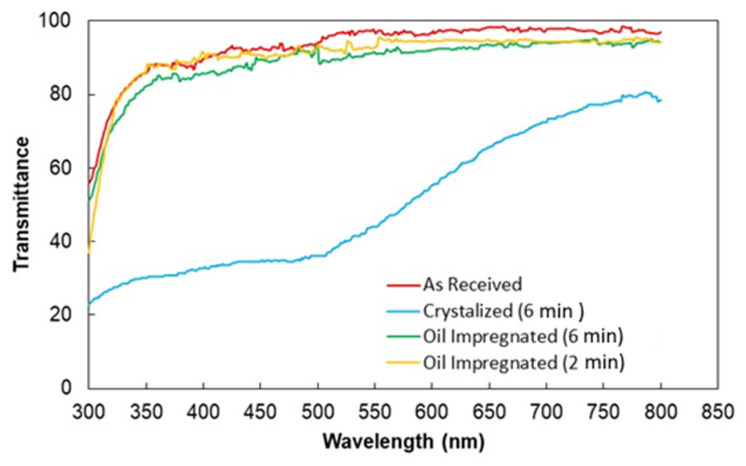
UV visible transmittance of treated surfaces prior and after oil impregnation. Optical transmittance of as received polycarbonate is also provided for comparison.

**Figure 9:**
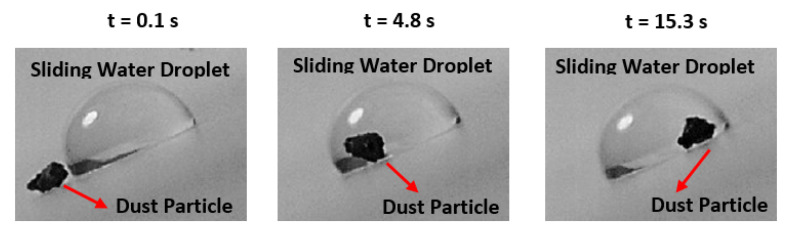
Optical images of sliding water droplet and dust particle over silicon oil impregnated surface. Red arrow shows the location of dust particle on the surface and after t = 4.8 s dust particle is picked up by the sliding water droplet.

UV visible transmittance of treated surfaces was carried out and the transmittance data are shown in Figure 8. The optical transmittance of the as-received sample is provided in the figure for comparison. Surface topology created after solution treatment gives rise to scattering and partial absorption of the incident optical radiation, which causes a reduction in transmittance. The transmittance reduction is almost similar for both treated surfaces. However, one of the methods to improve the UV visible transmittance of the treated samples is the oil impregnation of the treated surface, i.e., impregnation with a fluid having a similar refractive index of polycarbonate. Silicon oil is one of the good candidates in this regard. However, assessment of spreading of silicon oil over the treated surface is necessary to achieve uniform oil film thickness over the treated surface. In this case, the spreading factor of silicon oil ($S_{o-s}$) must remain greater than unity, i.e., $S_{o-s}>1$. The spreading factor of silicon oil on the treated surface can be expressed as [32]:

(5) $$S_{o-s}=\gamma_{pc}-\gamma_{pc-o}-\gamma_{o}$$

here, $\gamma_{pc}$ is surface free energy of polycarbonate, $\gamma_{pc-o}$ is interfacial tension between oil and treated surface, and $\gamma_{o}$ is surface tension of silicon oil. The treated surface free energy is determined previous as $\gamma_{pc}≅$ 36.2 mJ/m^2^ [15] and the silicon oil surface tension is 0.0187 N/m [33]. Moreover, the interfacial tension ($\gamma_{pc-o}$) can be obtained via Hemi-Wicking state [34]. This gives rise to:

(6) $$\gamma_{pc-o}=\gamma_{s}-\frac{\gamma_{o}cos\theta_{pc-o}}{f}$$

where, $cos\theta_{pc-o}$ is water contact angle of on smooth crystallized surface. The value of $\gamma_{pc-o}$ is estimated as 4.6 mN/m. Consequently, using the relation for the spreading factor, $S_{o-s}$ yields 12.9 mN/m. Since $S_{o-s}$ is greater than zero, silicon oil spreads over the treated surfaces. The optical transmittance of the silicon oil impregnated and treated surface is shown in Figure 8. The UV visible transmittance of the sample is improved significantly after the oil impregnation. The water droplet behavior on oil impregnated surface is also assessed using the high-speed recording data and the tracker program. The droplet does not roll over the oil impregnated surface but rather slides. Figure 9 shows optical images of sliding water droplet over the impregnated surface with and without dust particles. It is evident that the dust particle is picked up by the sliding water droplet. Hence, silicon oil impregnation improves the UV transmittance of the treated surface and facilitates the dust removal by a sliding droplet, which is true for both impregnated and treated surfaces. Therefore, for photovoltaic protective panels, a thin layer of oil film becomes essential over the crystallized surface for improved UV visible transmittance.

### 3.4. Life Cycle Analysis for Solution Crystallization of Surface

**Figure 10:**
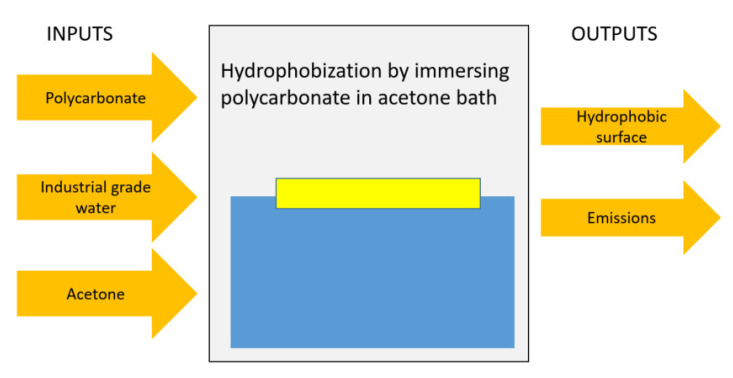
Boundary considered in life cycle analysis for hydrophobization of polycarbonate.

**Figure 11:**
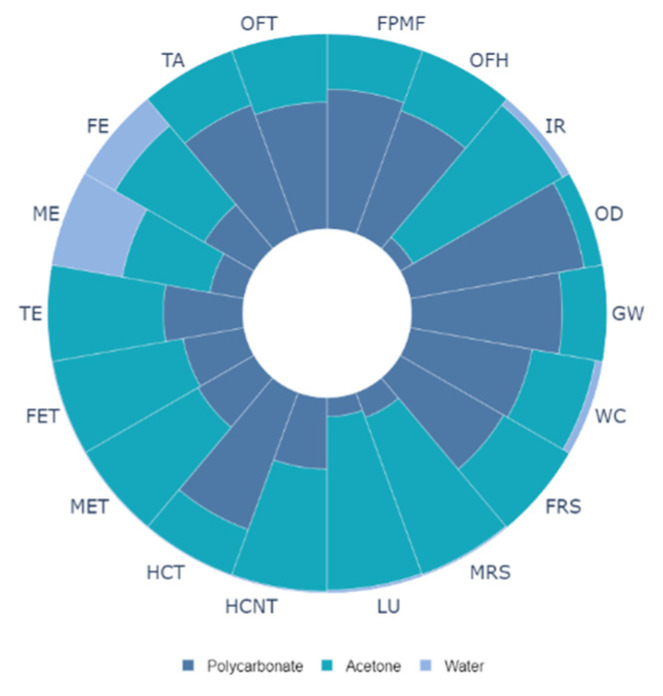
Normalized cradle-to-gate environmental profile for the hydrophobizing process. Here, GW is global warming, OD is stratospheric ozone depletion, IR is ionizing radiation, OFH is ozone formation, human health, FPMF is fine particulate matter formation, OFT is ozone formation, terrestrial ecosystems, TA is terrestrial acidification, FE is freshwater eutrophication, ME is marine eutrophication, TE is terrestrial ecotoxicity, FET is freshwater ecotoxicity, MET is marine ecotoxicity, HCT is human carcinogenic toxicity, HCNT is human non-carcinogenic toxicity, LU is land use, MRS is mineral resource scarcity, FRS is fossil resource scarcity, and WC is water consumption.

**Figure 12:**
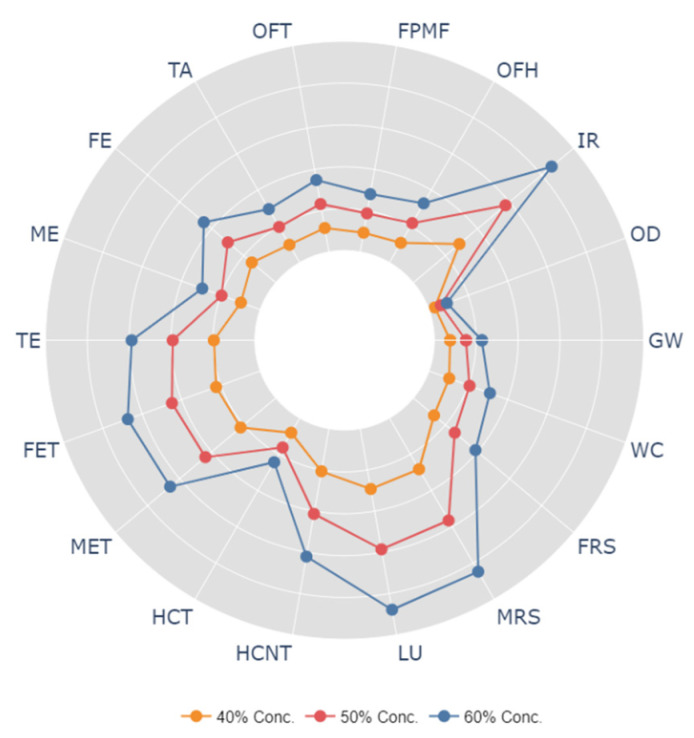
Sensitivity analysis for three different acetone concentrations. Here, GW is global warming, OD is stratospheric ozone depletion, IR is ionizing radiation, OFH is ozone formation, human health, FPMF is fine particulate matter formation, OFT is ozone formation, terrestrial ecosystems, TA is terrestrial acidification, FE is freshwater eutrophication, ME is marine eutrophication, TE is terrestrial ecotoxicity, FET is freshwater ecotoxicity, MET is marine ecotoxicity, HCT is human carcinogenic toxicity, HCNT is human non-carcinogenic toxicity, LU is land use, MRS is mineral resource scarcity, FRS is fossil resource scarcity, and WC is water consumption.

Life cycle analysis for solution crystallization of polycarbonate surface is carried out to evaluate the environmental impact of the one-step crystallization process. Figure 9 shows the system boundaries for cradle-to-gate analysis towards assessing the environmental impacts related to hydrophobization of polycarbonate wafers. The findings of the life cycle impacts for the hydrophobization process are summarized in Table 2. It is worth mentioning that the environmental impacts are expressed utilizing ReCiPe impact assessment at midpoint levels [35], and 18 impact categories are used in the analysis. The impact categories and their respective units are also listed in Table 2. It can be observed from Table 2 that producing 1 m^2^ of the hydrophobic surface gives rise to the global warming potential equal to 0.0025 kg of CO_2_, terrestrial acidification potential equal to 0.0013 kg of SO_2_, and human carcinogenic toxicity equal to 0.155 kg of 1,4–DCB. These values seem to be not very significant as compared to the other processes such as laser treatment. However, as the solution treatment process is industrialized, these values can increase manifold. In order to understand the contribution of each material (polycarbonate and solution) used in the crystallization process, normalized impacts for each material were computed and plotted in Figure 10. It is worth mentioning that solution consists of acetone and water. Each impact indicator is normalized and total indicator for each category is set at 100%. In the solution crystallization process, both polycarbonate and acetone are the main contributors to all impact categories (Figure 10). For example, polycarbonate contributes 77% towards global warming, 90% towards ozone depletion, and 71% toward fine particulate matter formation. In addition, acetone is the major contributor to ionizing radiation (88%), freshwater ecotoxicity (68%), and mineral resource scarcity (85%). Use of industrial grade water is more prominent in freshwater eutrophication (18%) and marine ecotoxicity (75%). In order to further analyze the relationship between the parameters related to the solution crystallization process and the impact categories, the sensitivity analysis was carried out. Three scenarios were considered for sensitivity analysis; in which case, the acetone concentration was considered to be increasing to 40%, 50%, and 60% in the solution bath. The findings of the sensitivity analysis are shown in Figure 11. The figure shows the percentage difference in each impact category with respect to the baseline concentration. It is evident from Figure 12 that changing the solution bath parameters can potentially change the cradle-to-gate life cycle emissions. It is obvious that increasing acetone concentration can lead to higher life cycle impacts. For example, increasing acetone concentration from 30% to 40% can increase the impact categories of global warming by 7%, ionizing radiation by 30%, freshwater eutrophication by 15%, and human carcinogenic toxicity by 8%. The increase of the environmental impact parameters with acetone was found to be in a linear form.

## 4. Conclusions

Surface texture topology consisting of spherules and fibrils were created through solution treatment of polycarbonate wafers. Wetting states of the treated surfaces were evaluated and the effect of solution treatment duration on the texture topology was analyzed. The resulting texture characteristics and water rolling droplet motion towards dust removal were assessed. The UV visible transmittance of the treated surfaces reduces because of the texture topology. Oil impregnation was utilized over the treated surfaces to improve the optical transmittance of the treated samples. The life cycle analysis and environmental impacts were carried out for the solution treatment process towards crystallizing polycarbonate surface. Findings revealed that solution treatment of polycarbonate results in hydrophobic states on the surface. The resulting surface texture topology has micro and nano-size spherules and fibrils. Increasing treatment duration to 6 min increases the spherules′ height and reduces the spaces between them. This causes increased water droplet contact angle (144 ± 3°) and high contact angle hysteresis. The pinning force of the water droplet becomes larger for 6 min treated surfaces than that of 2 min, which is because of large contact angle hysteresis, i.e., it is 22 ± 3° for 2 min treatment and 28 ± 3° for 6 min treatment. The amount of dust mitigated by the rolling droplet from 6 min treated surface is less than its counterpart of 2 min treated surface. This is because the high spherules height prevents droplet fluids from reaching the treated surface. Surface topology created during solution treatment lowers optical transmittance. Oil impregnation over the treated surface improves UV visible transmittance of the samples. However, droplet motion changes from rolling to sliding on the oil-impregnated surface. In this case, the rolling droplet velocity becomes larger than the sliding droplet velocity. Nevertheless, the dust particles can be picked up by the sliding water droplet from the oil-impregnated surface. In addition, the crystalized surfaces can be used in various applications for which the surface hydrophobicity is obliged without the requirement of optical transmittance. The results of the life cycle analysis demonstrate that both polycarbonate and acetone used in the crystallization process contributed the most towards 18 environmental impact categories considered in the current study. The life cycle analysis for the solution treatment adds new knowledge to the production of crystallized polycarbonate surfaces and helps improve the sustainability of the hydrophobization process.

## Figures and Tables

**Table 1:** Contact angles measurement for as received and crystallized polycarbonate surface after 6 and 2 min treatment durations. The contact angle and hysteresis presented in the table provides the average values after 12 measurements. ±3° is related to experimental errors due to droplet spreading.

	Angle (Degrees)	Hysteresis (Degrees)
As received	85 ± 3°	45 ± 3°
Crystalized PC (6 min)	144 ± 3°	28 ± 3°
Crystalized PC (2 min)	138 ± 3°	22 ± 3°

**Table 2:** LCA results for hydrophobization of polycarbonate via solution crystallization while using 30% acetone in the solution.

Impact Category	Symbol	Unit	Impact Value
Global warming	GW	kg CO_2_ eq	0.002564
Stratospheric ozone depletion	OD	kg CFC-11 eq	0.000105
Ionizing radiation	IR	kBq Co-60 eq	0.000137
Ozone formation, human health	OFH	kg NO_x_ eq	0.001785
Fine particulate matter formation	FPMF	kg PM2.5 eq	0.00081
Ozone formation, terrestrial ecosystems	OFT	kg NO_x_ eq	0.002214
Terrestrial acidification	TA	kg SO_2_ eq	0.0013
Freshwater eutrophication	FE	kg P eq	0.00214
Marine eutrophication	ME	kg N eq	2.01 × 10^−5^
Terrestrial ecotoxicity	TE	kg 1,4-DCB	0.012569
Freshwater ecotoxicity	FET	kg 1,4-DCB	0.124233
Marine ecotoxicity	MET	kg 1,4-DCB	0.197215
Human carcinogenic toxicity	HCT	kg 1,4-DCB	0.155352
Human non-carcinogenic toxicity	HCNT	kg 1,4-DCB	0.021281
Land use	LU	m^2^a crop eq	4.31 × 10^−5^
Mineral resource scarcity	MRS	kg Cu eq	9.21 × 10^−8^
Fossil resource scarcity	FRS	kg oil eq	0.006773
Water consumption	WC	m^3^	0.000572

## Data Availability

The data presented in this study are available on request from the corresponding author.
